# Plasma oxalate and eGFR are correlated in primary hyperoxaluria patients with maintained kidney function—data from three placebo-controlled studies

**DOI:** 10.1007/s00467-020-04894-9

**Published:** 2021-01-30

**Authors:** Dawn S. Milliner, Pierre Cochat, Sally-Anne Hulton, Jerome Harambat, Ana Banos, Bastian Dehmel, Elisabeth Lindner

**Affiliations:** 1 Division of Nephrology, Mayo Clinic, 200 First St. SW, Rochester, MN 55905 USA; 2 Centre de Référence des Maladies Rares Néphrogones, Hospices Civils de Lyon & Université Claude-Bernard Lyon, Lyon, France; 3 Birmingham Women’s and Children’s Hospital NHS Trust, Birmingham, UK; 4 Department of Pediatrics, Bordeaux University Hospital, Bordeaux, France; 5 OxThera Intellectual Property AB, Stockholm, Sweden

**Keywords:** Primary hyperoxaluria, Plasma oxalate, eGFR, Chronic kidney disease, Correlation, Clinical trials

## Abstract

**Background:**

In patients with primary hyperoxaluria (PH), endogenous oxalate overproduction increases urinary oxalate excretion, leading to compromised kidney function and often kidney failure. Highly elevated plasma oxalate (Pox) is associated with systemic oxalate deposition in patients with PH and severe chronic kidney disease (CKD). The relationship between Pox and estimated glomerular filtration rate (eGFR) in patients with preserved kidney function, however, is not well established. Our analysis aimed to investigate a potential correlation between these parameters in PH patients from three randomized, placebo-controlled trials (studies OC3-DB-01, OC3-DB-02, and OC5-DB-01).

**Methods:**

Baseline data from patients with a PH diagnosis (type 1, 2, or 3) and eGFR > 40 mL/min/1.73 m^2^ were analyzed for a correlation between eGFR and Pox using Spearman’s rank and Pearson’s correlation coefficients. Data were analyzed by individual study and additionally were pooled for Studies OC3-DB-02 and OC5-DB-01 in which the same Pox assay was used.

**Results:**

A total of 106 patients were analyzed. A statistically significant inverse Spearman’s correlation between eGFR and Pox was observed across all analyses; correlation coefficients were − 0.44 in study OC3-DB-01, − 0.55 in study OC3-DB-02, − 0.51 in study OC5-DB-01, and − 0.49 in the pooled studies (*p* < 0.0064).

**Conclusions:**

Baseline evaluations showed a moderate and statistically significant inverse correlation between eGFR and Pox in patients with PH already at early stages of CKD (stages 1–3b), demonstrating that a correlation is present before substantial loss in kidney function occurs.

**Graphical abstract:**

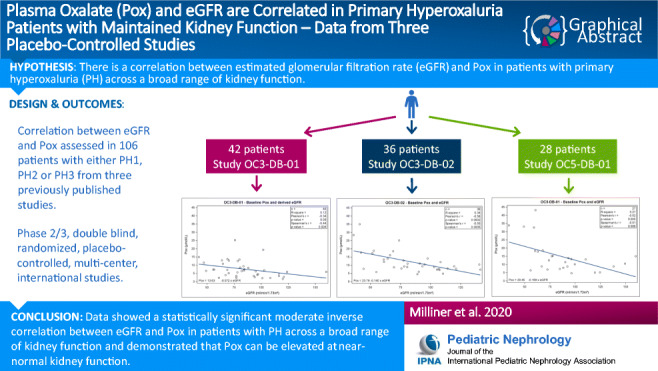

**Supplementary Information:**

The online version contains supplementary material available at 10.1007/s00467-020-04894-9.

## Introduction

Primary hyperoxalurias (PHs) are a group of rare genetic disorders caused by a range of defects in hepatic glyoxylate metabolic processes. Although the three identified forms of PH (PH1, PH2 and PH3) are associated with mutations in different genes (*AGXT*, *GRHPR*, and *HOGA1*, respectively), all are characterized by endogenous overproduction of oxalate, a byproduct of normal human metabolism that has low solubility [1–4]. Humans lack the enzymes required to metabolize oxalate, therefore it is excreted primarily via the kidneys in urine [5]. Patients with PH excrete high concentrations of oxalate in the urine, leading to formation of calcium oxalate (CaOx), which precipitates as crystals when urinary supersaturation occurs. Deposits of CaOx in the tubular lumen and renal parenchyma can result in nephrocalcinosis and urolithiasis. Internalization of CaOx crystals leads to inflammation, decreased kidney function, and often kidney failure [2, 6–8]. In chronic kidney disease (CKD) stages 3b–4, plasma oxalate (Pox) levels increase dramatically, ultimately depositing CaOx systemically in organ systems throughout the body in a potentially life-threatening process known as systemic oxalosis [9, 10]. Once kidney failure occurs, intensive dialysis regimens and eventually kidney transplantation, often in combination with a liver transplantation, are required. The most severe form of PH, type 1, accounts for approximately 80% of cases [1, 11, 12].

Identification of robust endpoints for clinical trials is crucial to permit earlier diagnosis and treatment of patients with PH before the more severe complications of the disease arise. Surrogate endpoints in clinical trials do not directly measure the clinical benefit of the treatment in question; rather, they can be useful as predictors of treatment efficacy, often by using biomarkers such as laboratory measurements or physical signs [10], which could be tied to a clinical benefit.

Given the rarity of the disease [1], recruitment of patients with PH to clinical trials is difficult and current treatment options are limited. In patients with PH and advanced CKD (stages 3b–5; estimated glomerular filtration rate (eGFR) < 45 mL/min/1.73 m^2^), highly elevated Pox is directly related to the pathophysiology of oxalosis, and substantial change in Pox may be used as a surrogate endpoint in a clinical setting [10]. There are, however, discrepancies in the literature regarding the relationship of Pox and eGFR in patients with earlier stages of CKD. Data from a recent study indicated that there is a relationship between Pox and eGFR even for patients with less advanced stages of CKD (stages 1–3; mean eGFR at baseline 111 mL/min/1.73 m^2^) [13]. In a registry of mostly US patients with PH, a clear inverse correlation between Pox and eGFR across a broad range of kidney function was observed [14]. Conversely, in a recent analysis of PH1 patients with stable kidney function from a European registry, no significant correlation between Pox and eGFR was detected [15].

In order to gain a better understanding of the potential association between Pox and eGFR in patients with PH and eGFR > 40 mL/min/1.73 m^2^, we investigated the correlation of these parameters using separate, and pooled, data from three randomized, placebo-controlled trials that have previously been published [16–18].

## Methods

### Studies

The objective of all three interventional studies was to evaluate the efficacy and safety of *Oxalobacter formigenes*, an anaerobic oxalate-degrading gut bacterium (Oxabact, OC3, or OC5, OxThera Intellectual Property AB, Sweden) [19–21]. *O. formigenes* is suggested to reduce the endogenous oxalate burden by promoting the removal of oxalate from the plasma into the intestine via passive and active flux processes [22, 23].

The current analysis was based on individual and pooled baseline data from three studies previously published which are briefly summarized as follows:

Study OC3-DB-01 (ClinicalTrials.gov Identifier: NCT00638703) was a phase 2/3, double-blind, randomized, placebo-controlled, multicenter, international study conducted in 42 patients with PH1 and PH2 between 2007 and 2008. Patients received OC3 formulation (enteric-coated capsule) or placebo twice daily for 24 weeks [16].

Study OC3-DB-02 (ClinicalTrials.gov Identifier: NCT01037231) was a phase 2/3, double-blind, randomized, placebo-controlled, multicenter, international study conducted in 36 patients with PH1 and PH2 between 2010 and 2011. Patients received OC3 formulation 500 mg (reconstituted buffer solution) or placebo twice daily for 24 weeks [17].

Study OC5-DB-01 (ClinicalTrials.gov Identifier: NCT02012985) was a phase 1/2, double-blind, randomized, placebo-controlled, multicenter, international study conducted in 28 patients with PH1, PH2 and PH3 between 2013 and 2015. Patients received OC5 formulation (enteric-coated capsule) or placebo twice daily for 8 weeks [18].

All studies were performed in accordance with good clinical practice (GCP) and the principles enshrined in the Declaration of Helsinki, and were approved by the relevant ethics committees. Patients were enrolled after providing written, informed consent and/or parental consent, depending on the age of the patient. The studies were designed, funded, and managed by OxThera Intellectual Property AB (Stockholm, Sweden).

### Inclusion/exclusion criteria

Study OC3-DB-01 included patients aged ≥ 5 years with urinary oxalate excretion > 1.0 mmol/1.73 m^2^/24 h and eGFR ≥ 50 mL/min/1.73 m^2^. Study OC3-DB-02 and study OC5-DB-01 included patients aged ≥ 2 years (≥ 5 years in the UK) with urinary oxalate excretion > 1.0 mmol/1.73 m^2^/24 h and eGFR ≥ 40 mL/min/1.73 m^2^.

### Estimated glomerular filtration rate

The eGFR equations used for the pediatric and adult populations, respectively, were Schwartz bedside 2009, eGFR = 0.413 × (height [cm]/(SCr) (where SCr is serum creatinine) [24] and MDRD 2007, eGFR = 175 × (SCr)^−1.154^ × (age)^−0.203^ × 1.212 (if black) × 0.742 (if female) [25].

### Plasma oxalate analysis

Blood samples were obtained at baseline and processed for determination of Pox. Plasma oxalate was analyzed using the “free” Pox method in study OC3-DB-01 and using the “total” Pox method in Studies OC3-DB-02 and OC5-DB-01. Free Pox was measured by the oxalate oxidase assay [26, 27] in which ultrafiltration of plasma removes proteins and circulating crystals, leaving only soluble oxalate for analysis. Normal values for free Pox with this assay are in the range of 1–3 μmol/L. Samples for free Pox determination were analyzed at the Mayo Central Laboratory for Clinical Trials, Rochester, USA.

Total Pox was measured using an isotope dilution mass spectrometric assay (gas chromatography with mass selective detection) [28]. The samples were first acidified to hydrolyze proteins, release bound CaOx and to dissolve CaOx crystals, and Pox was then extracted with ethylacetate prior to analysis. Normal values for total Pox with this assay are in the range of ≤ 7 μmol/L. Samples for total Pox determination were analyzed at the Academic Medical Center, University of Amsterdam, Netherlands.

### Statistical methods

Analyses of studies OC3-DB-01, OC3-DB-02, and OC5-DB-01 at baseline are presented individually. Pooled baseline data were also evaluated for Studies OC3-DB-02 and OC5-DB-01. Data from study OC3-DB-01 were not included in the pooled analysis because the study used a different Pox assay, which could have affected the correlation. Six patients were identified as having participated in more than one of the three trials; their data was therefore only included once, in study OC3-DB-02, when pooling data.

Descriptive statistics are provided as mean, median, standard deviation (SD), and range for continuous variables; frequency and percentage are provided for categorical variables. All values used were collected at baseline before study drug administration. Patients were classified into CKD stages, according to the National Kidney Foundation guidelines, based on their baseline eGFR; study inclusion criteria permitted recruitment of patients with CKD stages 1–3.

The association of eGFR with Pox was examined along with characteristics of normal distribution and linear relationship [29]. Based on indications of Pox and eGFR data being nonnormally distributed, the nonparametric Spearman’s rank correlation coefficient was primarily used providing an indication of the monotone association between eGFR and Pox. The Pearson’s product-moment correlation coefficient, measuring the strength of the linear relationship between eGFR and Pox, and the coefficient of determination *R*^2^ (amount of variance in eGFR explained by Pox) were calculated as supplementary analyses. Scatterplots including the linear regression line, corresponding regression equation and, for the pooled data, a nonparametric smooth curve (local polynomial regression) were generated, capturing the general data trend based on local estimated scatterplot smoothing (LOESS) procedure fitting [30]. The data included in our analyses satisfied the independence assumption when evaluating the regression and the Pearson’s product-moment linear correlation coefficient. Results were not adjusted for multiple testing. All statistical analyses were conducted using SAS software (version 9.4; SAS Institute, Cary, NC, USA).

## Results

### Study population

A total of 106 patients were included: 42 patients from study OC3-DB-01, 36 patients from study OC3-DB-02, and 28 patients from study OC5-DB-01 (Table 1). Primary hyperoxaluria type 1 accounted for > 83% of diagnoses in all three studies; only one patient had a diagnosis of PH3. Median time since diagnosis was 5.7–9.0 years across studies. Most patients in each study had CKD stages 1 or 2: 36 patients (85.7%) in study OC3-DB-01, 29 patients (80.5%) in study OC3-DB-02, and 27 patients (85.7%) in study OC5-DB-01. No major differences in sex and age were found between studies, and the majority of all study patients were recorded as Caucasian or not African American.

**Table 1:** Demographic and baseline characteristics

	OC3-DB-01 (*N* = 42)	OC3-DB-02 (*N* = 36)	OC5-DB-01 (*N* = 28)
Age (years)			
Mean	14.1	18.4	14.5
SD	6.7	14.5	5.6
Median	13.0	15.0	14.5
Min, Max	6, 39	3, 62	3, 27
Sex (*n* (%))			
Male	19 (45.2)	19 (52.8)	15 (53.6)
Female	23 (54.8)	17 (47.2)	13 (46.4)
Race (*n* (%))			
Caucasian	35 (83.3)	35 (97.2)	N/A
Asian	5 (11.9)	N/A	N/A
Other	2 (4.8)	1 (2.8)	N/A
African American/Black	0	N/A	1 (3.6)
Not African American	N/A	N/A	27 (96.4)
PH type (*n* (%))			
Type 1	35 (83.3)	31 (86.1)	26 (92.9)
Type 2	7 (16.7)	5 (13.9)	1 (3.6)
Type 3	N/A	N/A	1 (3.6)
Time since diagnosis (years)^a^			
Mean	8.5	9.5	9.0
SD	5.9	10.0	5.3
Median	9.0	5.7	8.3
Min, Max	− 0.1^b^, 23.4	− 0.1^c^, 41.6	1.3, 19.6
CKD stage (n(%))			
Stage 1	15 (35.7)	16 (44.4)	12 (42.9)
Stage 2	21 (50.0)	13 (36.1)	12 (42.9)
Stage 3a	4 (9.5)	3 (8.3)	3 (10.7)
Stage 3b	2 (4.8)	4 (11.1)	1 (3.6)
eGFR (mL/min/1.73 m^2^)			
Mean	83.4	83.8	86.6
SD	24.8	27.5	30.5
Median	80.3	80.8	81.3
Min, Max	42.4, 160.0	36.1, 141.4	34.3, 159.9
Pox (μmol/L)^d^			
Mean	7.5^e^	12.1	14.7^f^
SD	5.1^e^	6.6	9.9^f^
Median	6.5^e^	9.9	12.5^f^
Min, Max	1.6, 25.2^e^	4.5, 35.0	4.6, 43.1^f^
Urinary oxalate (mmol/1.73 m^2^/24 h)^g^			
Mean	1.77	1.80	1.74
SD	0.59	0.50	0.59
Median	1.59	1.79	1.62
Min, Max	1.02, 3.13	1.05, 2.75	0.90, 3.08
Urinary oxalate per creatinine ratio (mmol/mol)			
Mean	184.4	179.6	174.0
SD	87.2	70.0	94.5
Median	160.3	178.8	146.7
Min, Max	65.3, 451.8	80.0, 415.0	55.5, 466.7

*eGFR* Estimated glomerular filtration rate; *Max* maximum; *Min* minimum; *Pox* plasma oxalate; *SD* standard deviation

^a^Date of written informed consent minus date of diagnosis

^b^One subject provided written informed consent but was kept in screening until a definitive primary hyperoxaluria diagnosis was made

^c^One subject had a primary hyperoxaluria diagnosis two days after randomization; the patient was previously diagnosed before randomization, however, the official diagnosis was lost between physicians

^d^Free plasma oxalate was calculated in study OC3-DB-01, while total plasma oxalate was calculated in studies OC3-DB-02 and OC5-DB-01

^e^*N* = 40

^f^*N* = 27

^g^Urinary oxalate was calculated without centrifuging in study OC5-DB-01

### Estimated glomerular filtration rate, plasma oxalate, and urinary oxalate

There were no notable differences in median (range) eGFR reported across studies at baseline (80.3 mL/min/1.73 m^2^ (42.4–160.0) in study OC3-DB-01, 80.8 mL/min/1.73 m^2^ (36.1–141.4) in study OC3-DB-02 and 81.3 mL/min/1.73 m^2^ (34.3–159.9) in study OC5-DB-02). Median (range) Pox was lower in study OC3-DB-01 at 6.5 μmol/L (1.6–25.2) than in studies OC3-DB-02 and OC5-DB-01 (9.9 μmol/L (4.5–35.0) and 12.5 μmol/L (4.6–43.1), respectively) at baseline. The overall range for individual Pox values was 1.6–43.1 μmol/L for the populations across all three studies. There were no differences in baseline urinary oxalate between the three studies; median (range) baseline urinary oxalate was 1.59 mmol/1.73 m^2^/24 h (1.02–3.13) in study OC3-DB-01, 1.79 mmol/1.73 m^2^/24 h (1.05–2.75) in study OC3-DB-02, and 1.62 mmol/1.73 m^2^/24 h (0.90–3.08) in study OC5-DB-01. Similarly, there were no differences in baseline urinary oxalate per creatinine ratio values between the three studies; median (range) baseline urinary oxalate per creatinine ratio was 160.3 mmol/mol (65.3–451.8) in study OC3-DB-01, 178.8 mmol/mol (80.0–415.0) in study OC3-DB-02, and 146.7 mmol/mol (55.5–466.7) in study OC5-DB-01.

### Correlation between plasma oxalate and estimated glomerular filtration rate

**Fig. 1:**
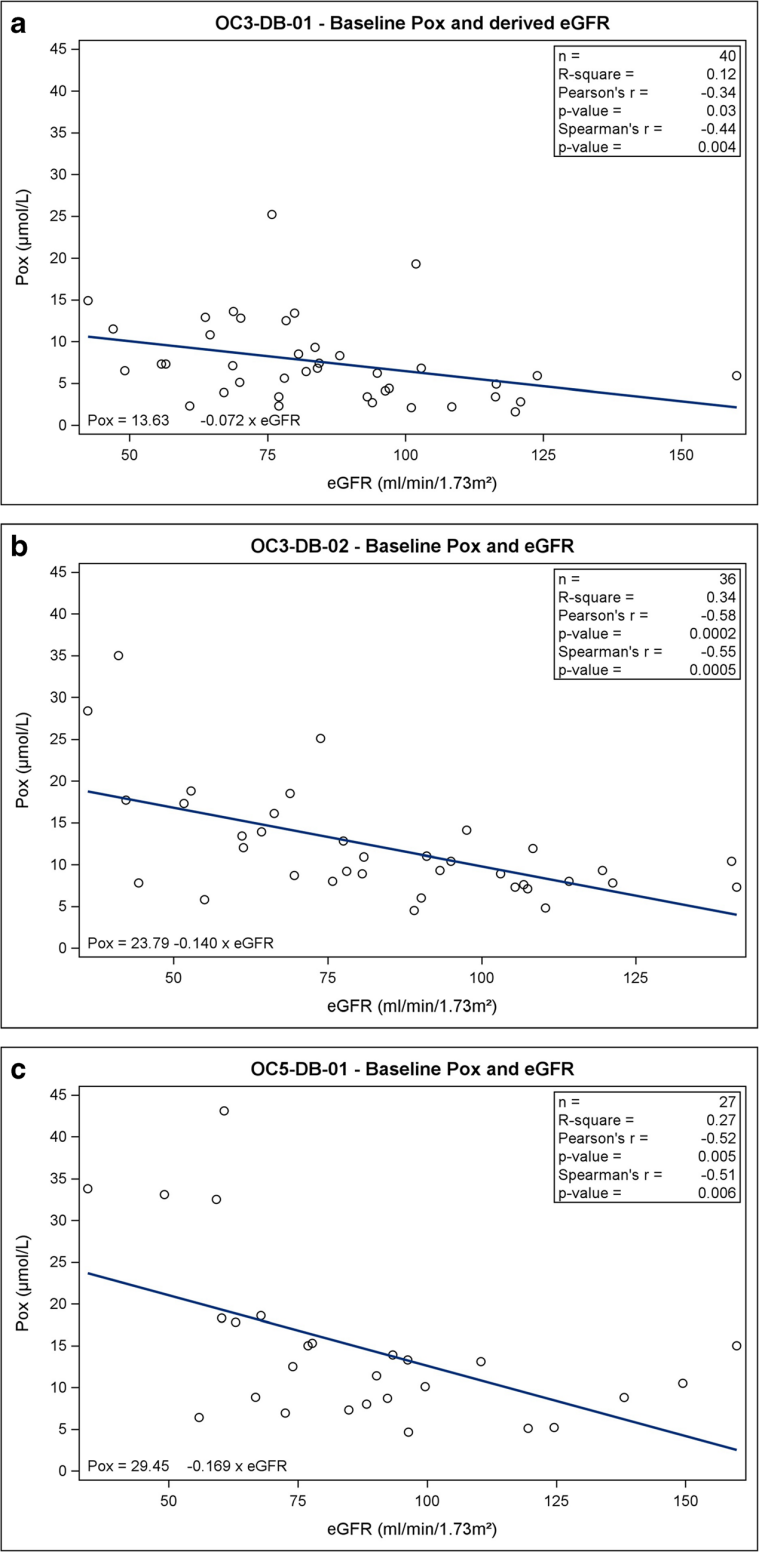
Plasma oxalate (Pox) vs. estimated glomerular filtration rate (eGFR). Individual values of Pox and eGFR in patients with both values recorded at baseline in **a** study OC3-DB-01 (n = 40), **b** study OC3-DB-02 (*n* = 36), and **c** study OC5-DB-01 (*n* = 27). Baseline Pox was measured using the free Pox method (study OC3-DB-01) or total Pox method (studies OC3-DB-02 and OC5-DB-01). Pox = plasma oxalate. eGFR = estimated glomerular filtration rate

**Fig. 2:**
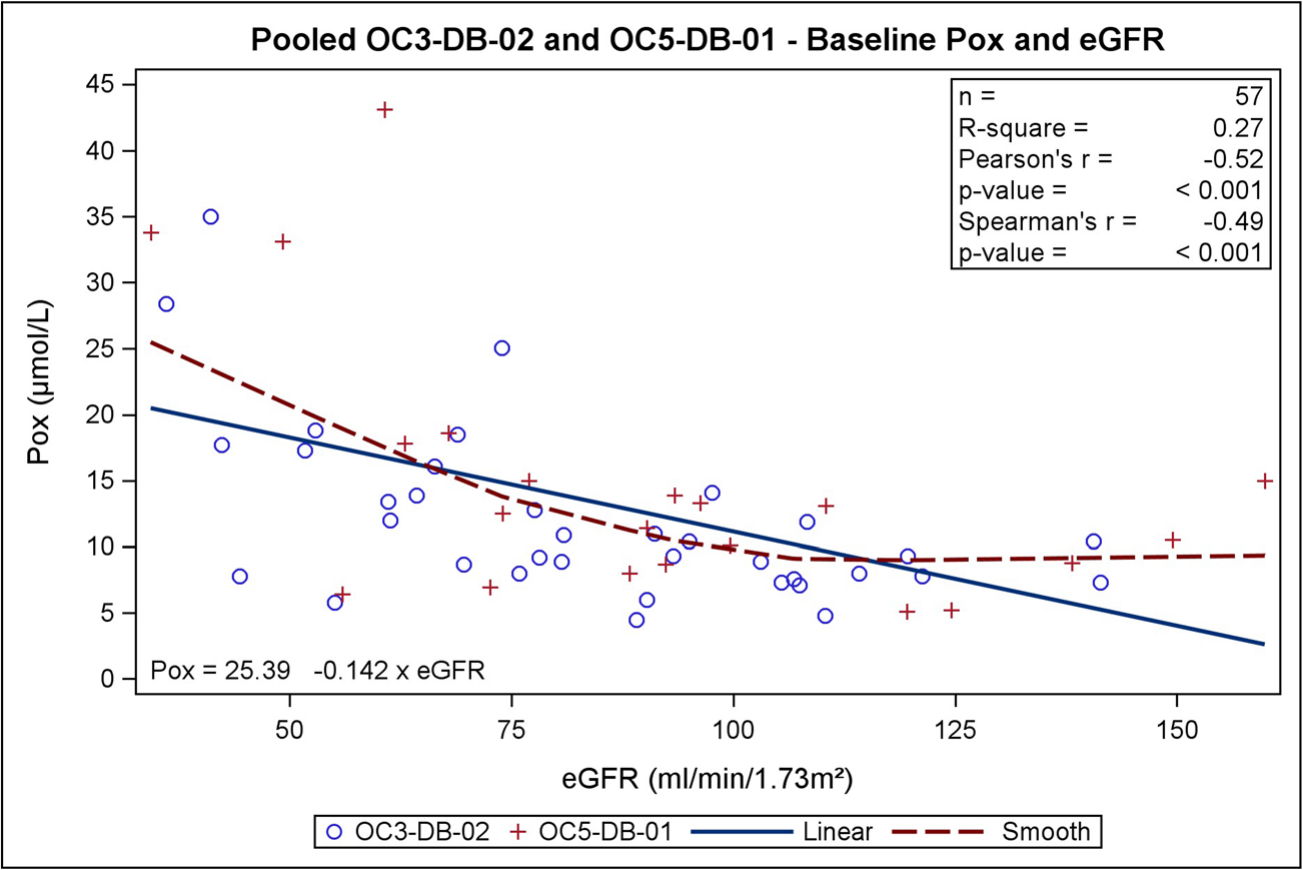
Pooled plasma oxalate (Pox) vs. estimated glomerular filtration rate (eGFR): Studies OC3-DB-02 and OC5-DB-01. Pooled individual values of Pox and eGFR in patients with both values recorded at baseline (*n* = 57) in studies OC3-DB-02 and OC5-DB-01. Six patients identified as participating in both studies were included only once, in study OC3-DB-02, when pooling data. Baseline Pox was measured using the total Pox method. Pox = plasma oxalate. eGFR = estimated glomerular filtration rate

Scatter plots are presented displaying the relationship between Pox and eGFR in study OC3-DB-01 (Fig. 1a), study OC3-DB-02 (Fig. 1b), and study OC5-DB-01 (Fig. 1c). A moderate and statistically significant inverse correlation between eGFR and Pox was observed (*p* < 0.05) in each of the three studies (Table 2), with Spearman’s correlation coefficients of − 0.44 in study OC3-DB-01, − 0.55 in study OC3-DB-02, and − 0.51 in study OC5-DB-01. Pooled data analyses from studies OC3-DB-02 and OC5-DB-01 (Fig. 2) also resulted in a statistically significant inverse correlation (*p* = 0.0001) between Pox and eGFR when analyzing linear regression, with a Spearman’s correlation coefficient of − 0.49. A statistically significant nonlinear relationship was also observed when local polynomial regression was used (Fig. 2; smooth line); the curve was steeper at eGFR values less than approximately 100 mL/min/1.73 m^2^ than those above.

**Table 2:** Correlation between Pox and eGFR

Study	Correlation^a^	*p* value
OC3-DB-01	− 0.44	0.0042
OC3-DB-02	− 0.55	0.0005
OC5-DB-01	− 0.51	0.0064
Pooled: OC3-DB-02 and OC5-DB-01	− 0.49	0.0001

^a^Spearman’s rank correlation coefficients are presented

## Discussion

Our analysis of baseline (predose) Pox and eGFR data from three randomized, placebo-controlled trials demonstrates a statistically significant inverse moderate correlation between eGFR and Pox in PH patients with CKD stages 1–3b. The correlation between Pox and eGFR has generally been thought to occur only in patients with advanced stages of CKD (stages 3b–5) [14, 31]. Previous reports [1] suggested that as a result of the better ability of kidneys to excrete excess oxalate at higher eGFR levels, Pox and eGFR may not correlate well in early stages of CKD. In contrast, the correlation of eGFR and Pox in the population reported in this analysis indicates that elevations in Pox are observed before substantial loss in kidney function has occurred. Thus, Pox may be a factor in the development of kidney damage, and could possibly be considered as a leading indicator in itself. Data from the pooled analysis suggests that the relationship between Pox and eGFR is nonlinear across the range of eGFR values assessed; the observed curve steepens when eGFR approaches and falls below 100 mL/min/1.73 m^2^.

In the general population, Pox concentrations are often too low to quantify, however our data suggest that in patients with PH, Pox is quantifiable even when eGFR is preserved, supporting its potential use as a biomarker [13, 14, 27]. The observed correlation between Pox and eGFR at higher eGFR ranges may support the utilization of Pox as a clinical trial endpoint facilitating determination of efficacy in future PH intervention studies in patients with CKD stages 1–3b. This conclusion is echoed by a recent consensus paper which supported the use of elevated Pox levels in patients with compromised kidney function (CKD stages 3b–5) as a likely surrogate endpoint for treatment efficacy, as well as proposing Pox as a causal factor for nephrocalcinosis and kidney stones in patients with preserved kidney function (CKD stages 1–3a) [10].

This is the first analysis to demonstrate a consistent correlation between Pox and eGFR using data from several randomized, placebo-controlled studies; an appropriate study design is paramount to acquiring high quality data, especially when the data are necessary to drive treatment advancement in rare diseases. While registry studies have provided significant information in PH, and remain a valuable tool in analyzing characteristics in patient populations, methodologies used are of importance in interpretation of results. For example, Pox concentrations in registry studies can be influenced by differences in methods used for sample handling, sample preparation, and analysis of Pox samples. High intra- and interlaboratory variability in reported values can therefore occur due to this lack of assay standardization, ultimately affecting diagnostic value and evaluation of treatment efficacy [32]. A study comparing Pox analysis methods used in patients with PH noted poor agreement in Pox reporting between six laboratories [33]. In the current analysis, we pooled data only from studies OC3-DB-02 and OC5-DB-01 because both used the same instructions for sample handling, the same Pox assay, and analyses were conducted at the same laboratory. One further advantage of using clinical trial data in our analyses is that the study protocols defined clearly the eligibility criteria, permitted concomitant medications and specific sampling time points, all of which can be a source of variability in registry data.

In addition to study design and the assays used for determination of Pox concentrations, there are additional confounding factors that can influence the relationship between Pox and eGFR, such as eGFR equations and intra- and individual patient differences. Other limitations could include the statistical methodology applied. Using primarily the nonparametric Spearman’s rank correlation coefficient in our study allowed us to assess the baseline data appropriately in the three studies we describe; this complied with assumption of data points independence, and normal distribution/linearity were not violated.

Though there is substantial intersubject variability in the progression and severity of disease, the effects of PH can be devastating. Improvements in diagnostic procedures and disease management have increased median survival of patients with PH1—cohort studies suggest a median kidney survival of between 24 and 33 years [11, 34, 35]—yet there is only a single, recently approved drug therapy for PH1 and no approved drug therapies for PH2 and PH3. Our analysis has certain limitations, chiefly that the low numbers of patients with a diagnosis of PH2 or PH3 did not permit sufficient statistical power to analyze the results by PH type. The majority of patients were Caucasian or not African American, highlighting the need for further research into patients of other racial and ethnic groups. Kidney function was estimated from the Schwartz and MDRD equations for practical reasons instead of formal measurement of GFR with, for instance, iohexol. Finally, the cross-sectional design of our study does not permit conclusions regarding a causal relationship between Pox concentration and change in eGFR. Despite these limitations, we demonstrate a statistically significant inverse correlation of moderate strength between Pox and eGFR in PH1 populations with CKD stages 1–3b. Other studies note the plausibility of Pox being on the causal pathway for kidney damage, that higher Pox concentrations can be a risk factor for kidney failure [10], and suggest that Pox concentration could potentially serve as a prognostic indicator of kidney function decline [13]. While our data cannot fully support these conclusions, the observed correlation at a single time point leads us to speculate that Pox concentration may at least serve as a marker for kidney function. This represents an area for further research.

In conclusion, our data from the baseline analysis of three randomized, placebo-controlled trials showed a statistically significant inverse correlation between eGFR and Pox in patients with PH across a broad range of kidney function (eGFR > 40 mL/min/1.73 m^2^ (CKD stage 1–3b)), and demonstrate that Pox can be elevated at kidney function traditionally regarded as normal. Prospective clinical studies are ongoing to evaluate change in Pox and its relationship to clinical outcome. Extensive research over the years has greatly advanced the development of desperately needed treatments for PH, and our study contributes to the growing body of evidence that could aid development of new strategies to assess clinical benefit in patients with PH.

## Supplementary Information

ESM 1 (PPTX 152 kb)

## Data Availability

The authors confirm that the data supporting the findings of this study are available within the article.
