# Supplementary material for: Plasma oxalate and eGFR are correlated in primary hyperoxaluria patients with maintained kidney function—data from three placebo-controlled studies
Source: Pediatr Nephrol. 2021 Jan 30;36(7):1785–93. doi: 10.1007/s00467-020-04894-9 (PMC8172484; doi:10.1007/s00467-020-04894-9)
Supplement: Supplementary file 1 — (PPTX 152 kb) [file 467_2020_4894_MOESM1_ESM.pptx]

## Slide 1
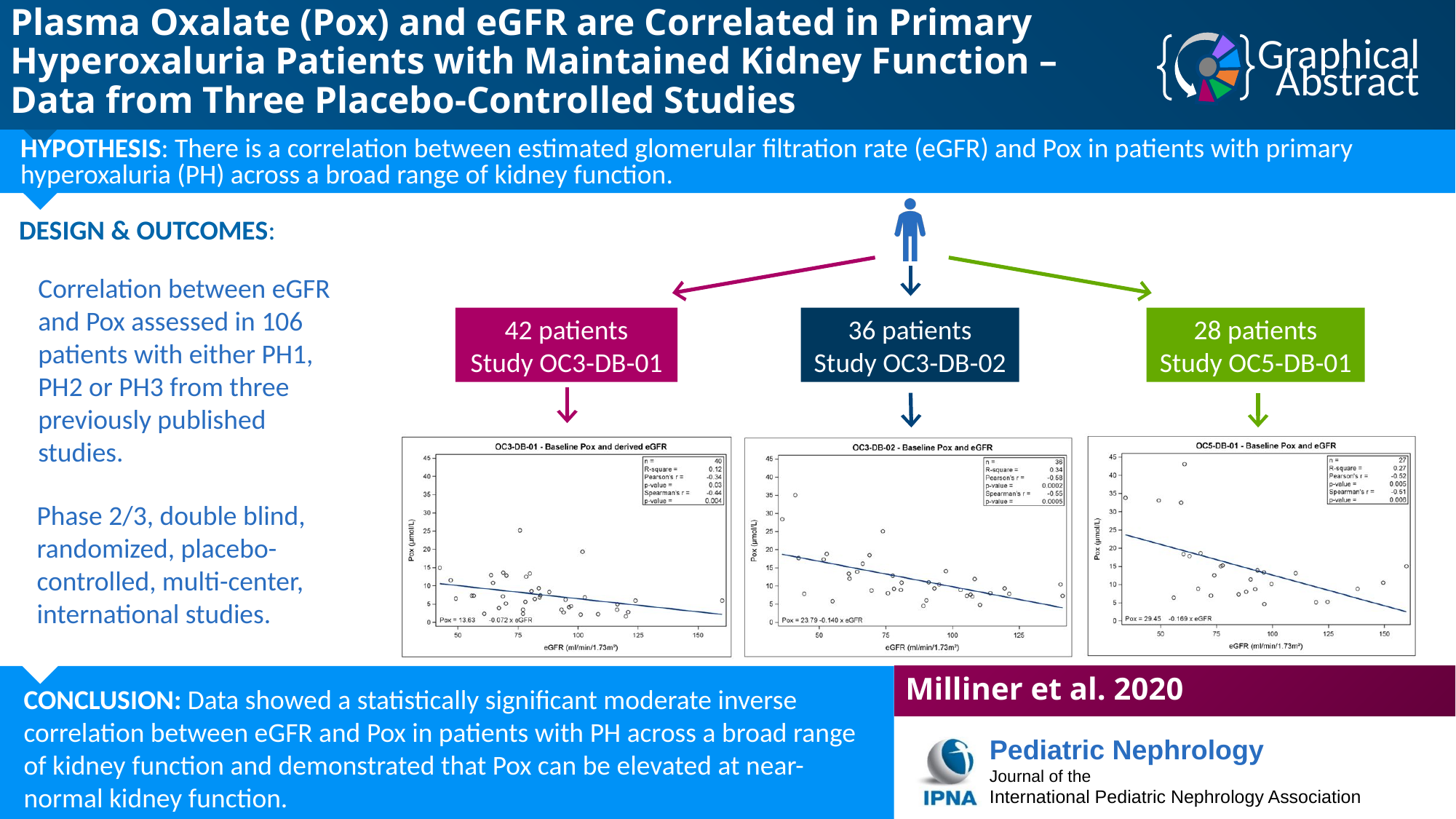

Plasma Oxalate (Pox) and eGFR are Correlated in Primary Hyperoxaluria Patients with Maintained Kidney Function – Data from Three Placebo‑Controlled Studies
HYPOTHESIS: There is a correlation between estimated glomerular filtration rate (eGFR) and Pox in patients with primary hyperoxaluria (PH) across a broad range of kidney function.
DESIGN & OUTCOMES:
Correlation between eGFR and Pox assessed in 106 patients with either PH1, PH2 or PH3 from three previously published studies.
42 patients
Study OC3‑DB‑01
36 patients
Study OC3‑DB‑02
28 patients
Study OC5‑DB‑01
Phase 2/3, double blind, randomized, placebo-controlled, multi-center, international studies.
Milliner et al. 2020
CONCLUSION: Data showed a statistically significant moderate inverse correlation between eGFR and Pox in patients with PH across a broad range of kidney function and demonstrated that Pox can be elevated at near-normal kidney function.
